# Diffraction based Hanbury Brown and Twiss interferometry at a hard x-ray free-electron laser

**DOI:** 10.1038/s41598-018-19793-1

**Published:** 2018-02-02

**Authors:** O. Yu. Gorobtsov, N. Mukharamova, S. Lazarev, M. Chollet, D. Zhu, Y. Feng, R. P. Kurta, J.-M. Meijer, G. Williams, M. Sikorski, S. Song, D. Dzhigaev, S. Serkez, A. Singer, A. V. Petukhov, I. A. Vartanyants

**Affiliations:** 1 Deutsches Elektronen-Synchrotron DESY, Notkestraße 85, D-22607, Hamburg, Germany; 2 National Research Tomsk Polytechnic University (TPU), Lenin Avenue 30, 634050, Tomsk, Russia; 3 SLAC National Accelerator Laboratory, 2575 Sand Hill Rd, Menlo Park, 94025 CA USA; 4 Van‘t Hoff Laboratory for Physical and Colloid Chemistry, Debye Institute for Nanomaterial Science, Utrecht University, Padualaan 8, 3584 CH, Utrecht, Netherlands; 5 European XFEL GmbH, Holzkoppel 4, D-22869, Schenefeld, Germany; 6 University of California San Diego, 9500 Gilman Dr., La Jolla, California, 92093 USA; 7 Laboratory of Physical Chemistry, Department of Chemical Engineering and Chemistry, Eindhoven University of Technology, P.O. Box 513, 5600 MB, Eindhoven, Netherlands; 8 National Research Nuclear University MEPhI (Moscow Engineering Physics Institute), Kashirskoe shosse 31, 115409, Moscow, Russia; 9 Present Address: European XFEL GmbH, Holzkoppel 4, D-22869, Schenefeld, Germany; 10 Present Address: Department of Physics, University of Konstanz, D-78457, Konstanz, Germany; 11 Present Address: NSLS-II, Brookhaven National Laboratory, 53 Bell Avenue, Upton, NY 11973-5000 USA; 12 Present Address: Department of Materials Science and Engineering, Cornell University, Ithaca, NY 14850 USA

## Abstract

X-ray free-electron lasers (XFELs) provide extremely bright and highly spatially coherent x-ray radiation with femtosecond pulse duration. Currently, they are widely used in biology and material science. Knowledge of the XFEL statistical properties during an experiment may be vitally important for the accurate interpretation of the results. Here, for the first time, we demonstrate Hanbury Brown and Twiss (HBT) interferometry performed in diffraction mode at an XFEL source. It allowed us to determine the XFEL statistical properties directly from the Bragg peaks originating from colloidal crystals. This approach is different from the traditional one when HBT interferometry is performed in the direct beam without a sample. Our analysis has demonstrated nearly full (80%) global spatial coherence of the XFEL pulses and an average pulse duration on the order of ten femtoseconds for the monochromatized beam, which is significantly shorter than expected from the electron bunch measurements.

## Introduction

X-ray free-electron lasers - being new ultrabright, femtosecond x-ray sources^1–3^ - have found an extensive application in a wide range of scientific fields such as structural biology^4,5^, solid density plasma^6^, matter under extreme conditions^7^, ultrafast photochemistry^8^, atomic physics^9^ and many others. One important aspect that makes XFELs substantially different from all other existing x-ray sources, is the degeneracy parameter, or an average number of x-ray photons in one state. While for present high-brilliance synchrotron sources this value is about 10^−2^, for XFEL sources it can reach such high values as 10^10^ ^10–14^. This makes XFEL sources similar to optical lasers, and implies the possibility of non-linear and quantum optics experiments, as was first suggested by Glauber^15^. This area in FEL science is just in its early stage of development^16–19^.

At the core of quantum optics experiments stays HBT interferometry^20,21^. Since its first demonstration, it was used, for example, to analyze nuclear scattering experiments^22^, to probe Bose-Einstein condensates^23–25^ or to study the effects of photon interaction in a nonlinear media^26^. HBT interferometry is especially well - suited to study the statistical behavior of XFELs due to their extremely short pulse duration. It allows extraction of both the spatial and temporal XFEL coherence properties^12,13,27^, as well as statistical information about the secondary beams and positional jitter^27^.

The coherence properties of an XFEL may often significantly affect its experimental performance. Several methods have been employed to study the spatial and temporal coherence of XFEL sources, such as Young’s interference experiment with double pinholes^10,11^ or with a stream of bimodal gold particles^28^, Michelson type interferometry^11,29–32^, and speckle contrast analysis^33–36^. HBT interferometry at XFEL sources was first proposed in ref.^37^ and, recently, experimentally realized at different XFEL sources^12,13,27,38^ (see for review^39^).

The basic idea of HBT interferometry^20,21^ is to determine the statistical properties of radiation from the normalized second-order intensity correlation function

1 $${g}^{\mathrm{(2)}}({{\bf{r}}}_{{\bf{1}}},{{\bf{r}}}_{{\bf{2}}})=\frac{\langle I({{\bf{r}}}_{{\bf{1}}})I({{\bf{r}}}_{{\bf{2}}})\rangle }{\langle I({{\bf{r}}}_{{\bf{1}}})\rangle \langle I({{\bf{r}}}_{{\bf{2}}})\rangle }$$

obtained by measuring the coincident response of two detectors at positions ***r***_**1**_ and ***r***_**2**_ (see for review^40^). In Eq. (1), *I*(***r***_**1**_), *I*(***r***_**2**_) are the intensities of the wave field in corresponding positions and the averaging denoted by brackets <...> is performed over a large ensemble of different realizations of the wave field, or different pulses in the case of XFEL radiation.

Here, we present results from HBT interferometry performed on the Bragg peaks originating from the scattering by colloidal crystals. Statistical changes of the XFEL beam structure from pulse to pulse lead to corresponding changes in the observed Bragg peaks intensity distribution. Therefore, fluctuating behavior of the Bragg peak intensity contains information about the statistical properties of the incident radiation typical for Self-Amplified Spontaneous Emission (SASE) XFELs^41^. This allowed us to extract information on statistical properties of the XFEL radiation during a diffraction experiment on colloidal crystals.

## Results

### The setup

**Figure 1:**
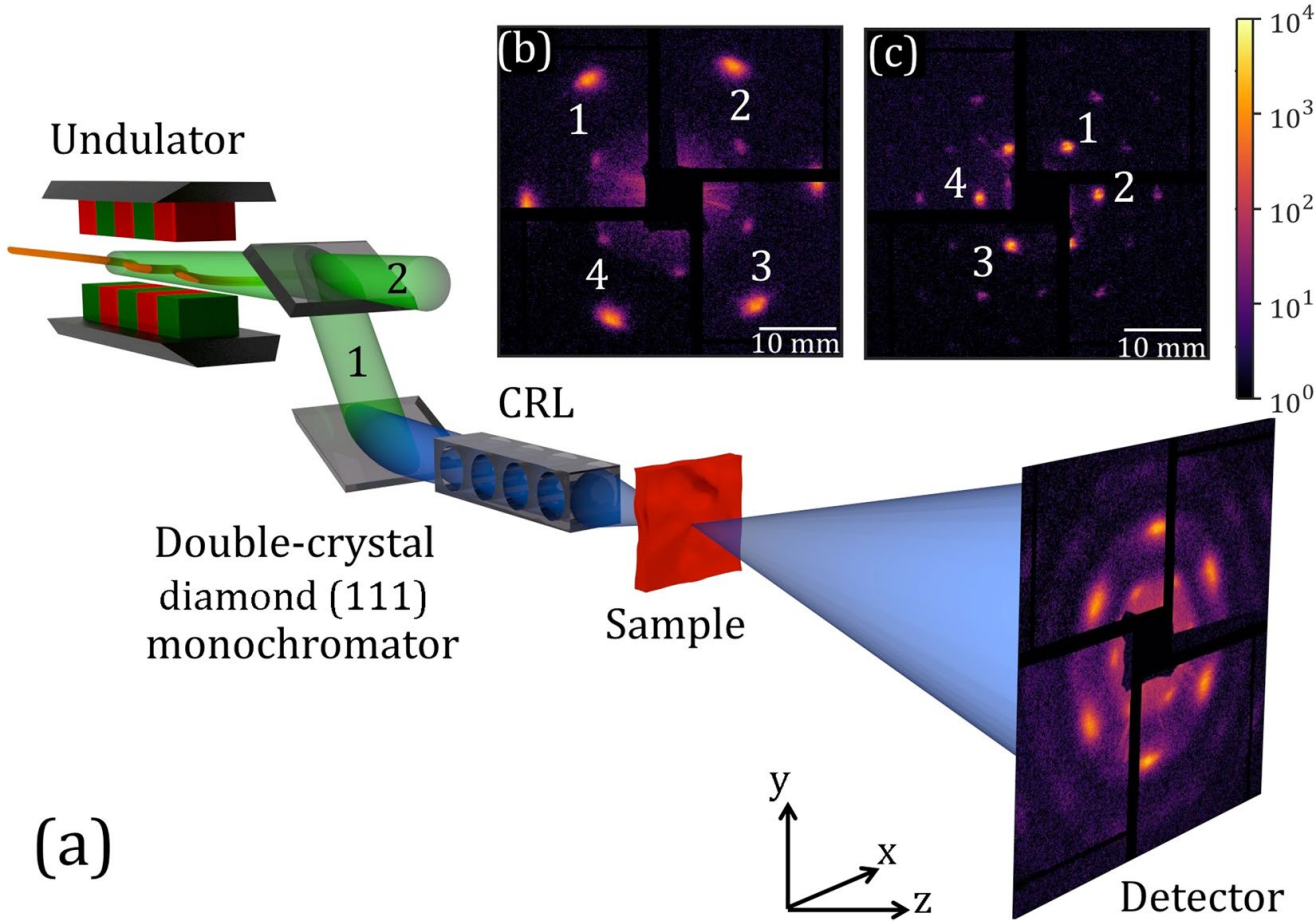
(**a**) Schematic layout of the experiment. LCLS radiation passes a double-crystal diamond (111) and is separated into diffracted (1) and transmitted (2) branches. The monochromatized radiation in the diffracted branch is focused on the sample by the compound refractive lenses (CRLs). Diffracted intensities are measured by the CSPAD detector positioned 10 m downstream from the sample. Central part of the typical diffraction patterns (shown in the log-scale) for a PS crystal with 160 nm sphere size (sample 1) (**b**) and a PS crystal with 420 nm sphere size (sample 2) (**c**). The peaks chosen for analysis are marked with white numbers.

The measurements were performed at Linac Coherent Light Source (LCLS) in Stanford, USA at the X-ray Pump-Probe (XPP) instrument^42^. We used the monochromatized radiation with a photon energy of 8 keV (1.55 Å) and relative energy bandwidth of 4.4⋅10^−5^. An expected pulse duration from electron bunch measurements was about 41–43 fs. The electron bunch length measurements were based on coherent edge radiation from the last dipole magnet of each compressor chicane and were calibrated in amperes of peak current using the transverse radio-frequency (RF) deflecting cavity^1^. A colloidal crystal sample was positioned vertically, perpendicular to the incoming XFEL beam, in the transmission diffraction geometry (see Fig. 1(a)). Two samples were investigated: polystyrene (PS) colloidal crystals composed of spheres with a diameter of 160 ± 3 nm (sample 1) and 420 ± 9 nm (sample 2). A megapixel x-ray Cornell-SLAC Pixel Array Detector (CSPAD) was positioned at  a distance *L* = 10 m downstream from the sample and used to record diffraction patterns (see, for experimental details, the methods section and ref.^43^). By using the CSPAD detector we substitute the two detector requirment for HBT measurements with two pixels of the same 2D detector.

### The measurements

**Figure 2:**
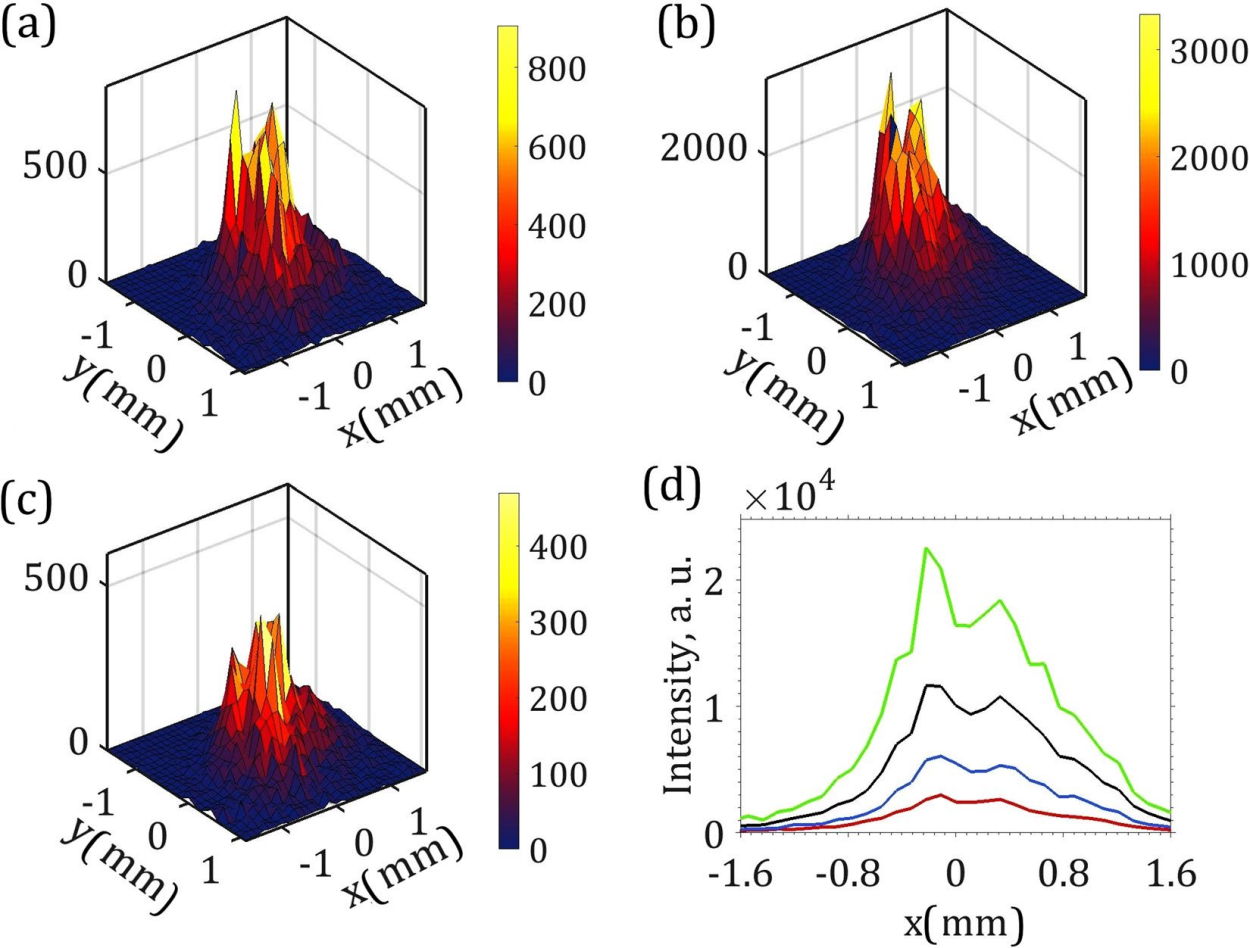
Single pulse intensities measured at the Bragg peak 4 for sample 2. (**a**–**c**) Typical 2D Bragg peak intensity distribution for different incoming pulses. (**d**) Projection of these intensities on the horizontal direction (intensity shown in (**a**) - blue curve, in (**b**) - green curve, and in (**c**) - red curve) and an average projected intensity for 50,000 pulses (black).

Typical diffraction patterns obtained in the experiment for our colloidal crystals are shown in Fig. 1(b) and (c). Bragg peaks with six-fold symmetry, originating from the hexagonal close-packed crystal structure, are clearly visible in this figure^44^. Due to a small beam size and large sample-detector distance, instead of the conventional sharp Bragg peaks, a comparably broad intensity distribution around each Bragg peak position is measured. Importantly, this intensity distribution depends not only on the crystal structure, but also on the incident pulse profile. Intensity distribution around Bragg peak 4 for sample 2 for three different incident pulses at the same position of the sample are shown in Fig. 2. It is well seen from this figure that Bragg peak profiles for each pulse have a complicated internal structure with additional sub-peaks. These sub-peaks have the same position from pulse to pulse but their relative intensity varies. Projection on the horizontal axis of the same Bragg peak intensities for three selected pulses, as well as an average projected intensity for all pulses, is shown in Fig. 2(d).

**Figure 3:**
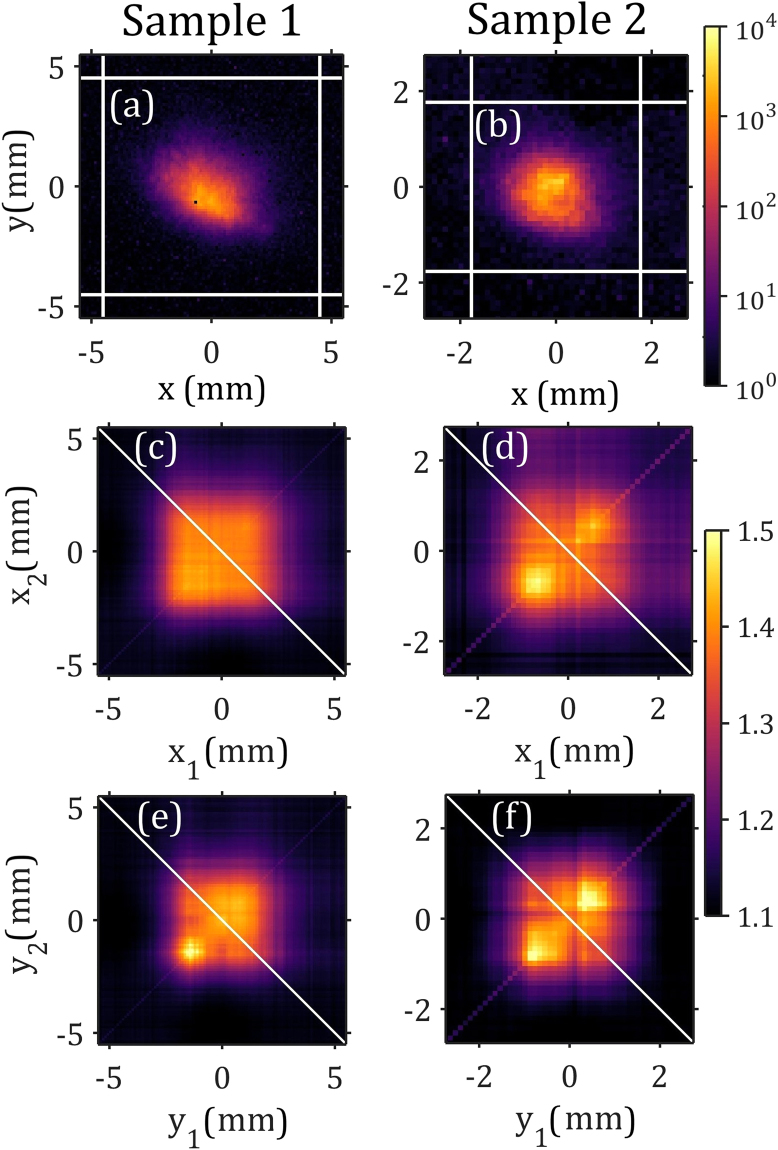
(**a**–**b**) Examples of average Bragg peak intensities (shown in log-scale) for sample 1 (**a**) and sample 2 (**b**) (peak 4 for both samples). (**c**–**f**) Intensity correlation functions *g*^(2)^(*x*_1_, *x*_2_) (**c**,**d**) and *g*^(2)^(*y*_1_, *y*_2_) (**e**,**f**) evaluated for the same peak 4 and corresponding to sample 1 (**c**,**e**) and sample 2 (**d**,**f**), respectively.

For correlation analysis we considered four Bragg peaks not obscured by detector gaps for each crystal (see Fig. 1(b) and (c)). Average intensities of the Bragg peak marked as 4 in Fig. 1(b) and (c) for both crystals are presented in Fig. 3(a) and (b). Projections of the selected Bragg peaks on the horizontal and vertical axes were then correlated and corresponding intensity correlation functions are presented in Fig. 3(c–f). The comparison of the intensity profiles with the intensity correlation functions reveals an interesting feature. While intensity profiles are not smooth and contain several sub-peaks reflecting the non-perfect structure of the colloidal crystals, the correlation functions are almost flat in a wide central region and then drop down fast to a background level, forming a square-type shape (see Supplementary Material for details). This is different from our earlier measurements at FELs^12,13,27^, when intensity correlation functions had been gradually decreasing with the distance between correlated positions.

Our experimental results allowed us to determine the degree of spatial coherence of LCLS radiation for hard x-rays (see Methods). Performing similar analysis as in refs^12,13,27^, we defined that the spatial degree of coherence for both samples and different peaks was, on average, about 0.90 ± 0.05 for each direction (horizontal and vertical), respectively (see Supplementary Material). As such, we obtained an estimate of global transverse coherence of the full beam to be 81%, which is in a good agreement with our previous observations at different XFEL facilities^10,11,27,39^.

HBT interferometry allowed us also to explore temporal properties of the beam (see Methods and Supplementary material). The functional dependence of the intensity correlation function as given by Eq. (2) in the Methods section allows one to study both the spatial and temporal statistical properties of the XFEL radiation by the HBT interferometry. An average pulse duration before the monochromator was estimated for each measured crystal and Bragg peak using equation (6) of the Method section. It is important to note that the effective pulse duration is extracted only from the part of the beam passing the monochromator. As such, it may be significantly shorter than that of the intrinsic beam generated by the undulator. The experimentally determined average pulse duration values lie in the range of 11–12 fs (see Supplementary Material for determined values), which is significantly shorter than initially expected (about 40 fs) from the electron bunch measurements. An excellent agreement between pulse durations determined for each crystal and Bragg peak suggests that the influence of the crystal structure variations on our results is insignificant.

To verify our findings, we determined pulse duration by a different approach based on the mode analysis of the radiation as suggested in ref.^45^. According to this approach, an average number of modes of radiation *M* is inversely proportional to the normalized dispersion of the energy distribution and can be connected to pulse duration (see Methods). One should be careful with such treatment, as energy jitter of the electron bunch may influence analysis of monochromator filtered radiation^38^. As such, in our further analysis we filtered the collected pulses bunch energies choosing only pulses with narrow energy distribution (see Supplementary materials for details). We determined the number of modes by fitting the intensity distribution at one of the Bragg peaks with Gamma distribution^45^ (see Supplementary Material for details). As a result, the number of longitudinal modes was *M* ≈ 2.3 ± 0.1 and reproducible between different runs. Substituting this number in Methods Eq. (7) gives, for the pulse duration, 11.5 ± 0.5 fs in an excellent agreement to previously determined values from the HBT interferometry.

The coherence time *τ*_*c*_ can be estimated from the bandwidth of the monochromator according to ref.^45^ as *τ*_*c*_ = (*π*)^1/2^/*σ*_*ω*_,where *σ*_*ω*_ is is the r.m.s. value of the monochromator bandwidth. The obtained value was about 7.5 fs for our monochromator settings^46^, which is only slightly shorter than the pulse duration. Therefore, x-ray pulses were effectively longitudinally coherent during the experiment.

### Simulations

**Figure 4:**
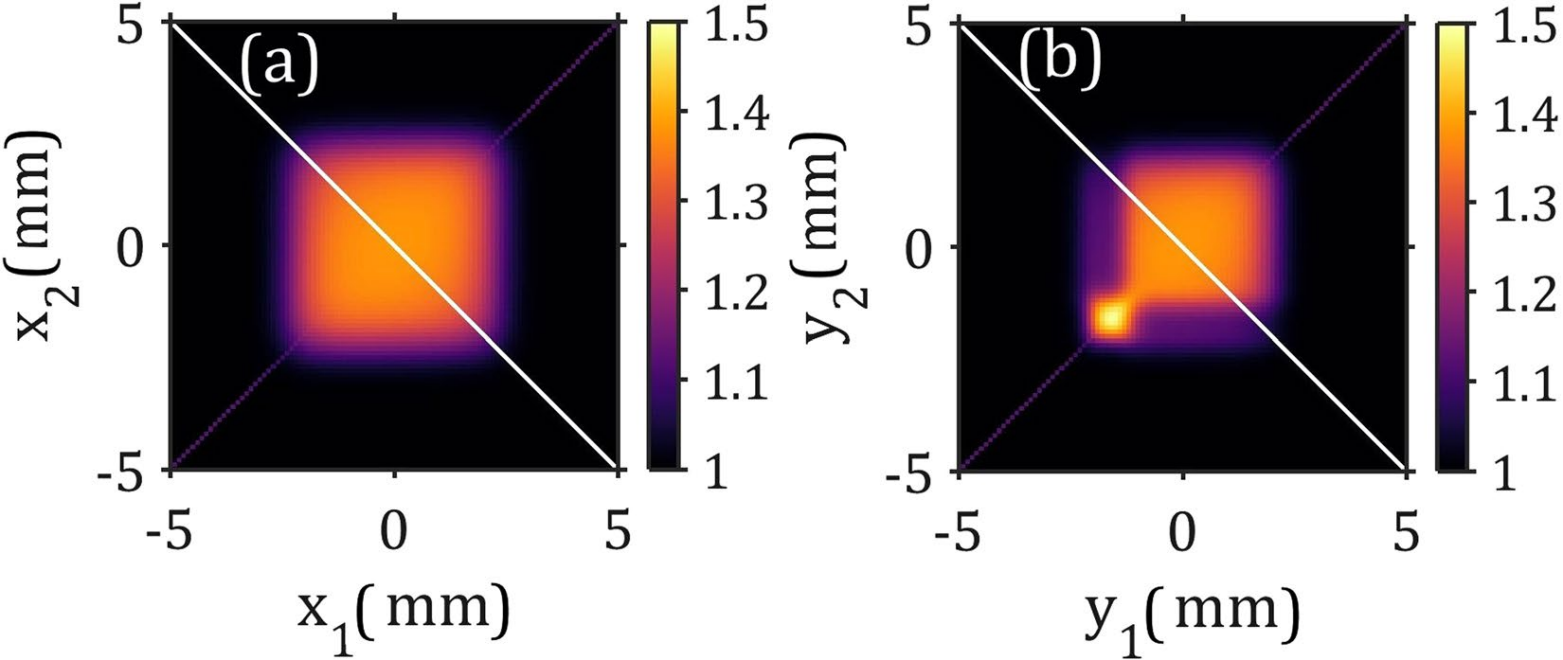
Simulated intensity correlation functions. (**a**) Single beam with a value of the spatial coherence length 10 mm which is much larger than the beam size (FWHM) of 1.6 mm and an additional background noise of 2% of the maximum intensity. (**b**) Strong main and a weak secondary beam with the same background noise. Secondary beam has 10% of the intensity of the main beam and is shifted by 1.5 mm in the vertical direction.

We were able to reproduce the unusual square-type shape of the intensity correlation functions observed in our experiment in simulations (see Fig. 4 and Supplementary Material for details). Two factors contribute to it: a coherence length larger than the beam size and additional detector noise. If a fluctuating background is simulated on the detector, it limits the field of view of the correlation function to the area where the intensity around the Bragg peak is larger than the detector noise. If the coherence length of the incident beam is a few times larger than the size of the beam, it leads to a relatively flat intensity correlation function.

We were also able to reproduce the appearance of a small area of higher contrast observed in Fig. 3(e). It can be simulated using the model of secondary beams introduced in^27^. A weak secondary beam with 10% of the primary beam intensity and shifted by 1.5 mm in the vertical direction (see for its characteristics Supplementary Material) leads to additional feature in the intensity correlation function. That is similar to that observed in the experiment (see Fig. 4(b)). The fact that the models based on the assumption of a chaotic source describe well the behavior of the intensity correlation function supports an assumption that LCLS, as a SASE XFEL, can be considered as a rather chaotic source (compare with^12,13^).

## Discussion

We demonstrated, experimentally, HBT interferometry at the XFEL facility in a diffraction mode by measuring at the Bragg peaks originating from scattering by colloidal crystals. This technique allowed us to extract information about spatial coherence and the temporal properties of the LCLS radiation directly from the diffraction patterns without additional equipment or dedicated measurements. We have determined a high degree of spatial coherence of the full XFEL beam - about 80% - which is in concordance with our previous measurements at XFELs. We have observed a coherence length much larger than the beam size. We have obtained pulse durations of 11–12 fs, which are significantly shorter than expected 41–43 fs in the operation regime of the LCLS used in our experiment. A similar inconsistency factor of about three between the expected and observed pulse duration has been observed earlier in another LCLS experiment^33^.

To explain the difference between these obtained values of the pulse duration with the results of the electron bunch measurements several factors should be taken into account. An estimate of the pulse duration from the electron bunch measurements is mainly based on the longitudinal size of the electron beam as an XFEL lasing medium. The electron beam size generally limits the maximum emitted hard x-ray pulse duration. The XFEL gain is very sensitive to various electron beam properties, such as beam emittance, electron current and energy spread, and orbit alignment inside an undulator. These properties vary along the electron beam, which may result in a relatively short core, providing significantly better gain, compared to the rest of the beam. Another possible explanation may be the filtering of the bunch with a strong chirp by the high-resolution monochromator^47^. It was proven experimentally with cross-correlation measurements^48^, that a 150 pC 50-fs long electron beam may only radiate a 14-fs long, 8.5 keV beam, which is comparable with our observations.

We also estimated coherence time after the high-resolution monochromator used in our experiment and obtained the value of 7.5 fs that is just slightly below the pulse duration. That means that LCLS pulses in our experiment were not only spatially but also temporally coherent, close to being Fourier-limited pulses.

We would like to emphasize that the presented approach is quite general and is not limited to the analysis of the diffraction patterns originating from colloidal crystals. Any other crystalline sample can be used, provided Bragg peaks are sufficiently broad to allow HBT measurement. This can be accommodated, for example, by a larger sample-detector distance, or implementing a set of compound refractive lenses (CRLs) in the beam diffracted from the sample.

Our measurements have demonstrated that statistical properties of XFEL radiation can be observed by measurements in diffraction. This may potentially lead to a completely new avenue in the field of quantum optics. Such quantum optics experiments as the exploration of non-classical states of light^49^, super-resolution experiments^50^, quantum imaging experiments^51,52^ or ghost imaging experiments^53–55^ may become possible at hard x-ray FEL sources in the near future. Finally, we foresee that diffraction-based HBT interferometry will become an important analytic tool in experiments at hard XFEL sources.

## Methods

### XFEL setup settings

LCLS was tuned to produce pulses with 3.3–3.7 mJ pulse energy, a bunch charge of 0.18 nC, and pulse repetition rate of 120 Hz. The double-crystal diamond (111) monochromator at LCLS with crystal thickness 100 *μ*m and 300 *μ*m split the primary x-ray beam into pink (transmitted) and monochromatic (diffracted) branches (see Fig. 1(a)). The beam size in the focus was 50 *μ*m full width at half maximum (FWHM) provided by CRLs. Focusing was necessary for the crystal structure studies. The number of photons in the focus was about 10^9^ ph/pulse and the experiment was performed in a non-destructive mode. This was confirmed by comparing diffraction patterns from the beginning and the end of the run. Series of x-ray diffraction patterns were recorded using the CSPAD megapixel x-ray detector, positioned at the distance *L* = 10 m downstream from the sample, consisting of 32 silicon sensors with the pixel size of 110 × 110 *μ*m^2^ covering an area of approximately 17 × 17 cm^2^. In order to exclude the influence of the electron energy jitter, only patterns corresponding to pulses with electron energies close to the mean value were selected (in total about 50,000, see Supplementary Material for details).

### Sample preparation

Colloidal crystal films were prepared from the PS colloids using the vertical deposition method^56^. The film consisted of 30–40 monolayers of spherical particles.

### Correlation function analysis

In our experimental geometry, we were in the Fresnel scattering regime (Fresnel number 1.7). It can be shown (see Supplementary Material for details) that in the general case of Fresnel scattering the intensity correlation function at a selected Bragg peak is given by the expression

2 $${g}^{\mathrm{(2)}}({\bf{Q}},{\bf{Q}}{\boldsymbol{^{\prime} }})=1+{\zeta }_{2}({\sigma }_{\omega }){|\mu ({\bf{Q}},{\bf{Q}}{\boldsymbol{^{\prime} }})|}^{2}\,\mathrm{.}$$

Here, the vector **Q** is related to a radius vector ***r***, measured from the center of the diffraction peak, by the relation **Q** = *k****r***/*L*, where *k* = 2*π*/*λ*, *λ* is the wavelength and *L* is the sample-detector distance. In the following we perform an evaluation in ***r***-space. The contrast function *ζ*_2_(*σ*_*ω*_) introduced in Eq. (2) is strongly dependent on the radiation bandwidth *σ*_*ω*_ and averaged pulse duration *T*. The spectral degree of coherence *μ*(**Q**,**Q′**) in Eq. (2) is defined as $\mu ({\bf{Q}},Q{\boldsymbol{^{\prime} }})=J({\bf{Q}},Q{\boldsymbol{^{\prime} }})/\sqrt{\langle I({\bf{Q}})\rangle }\sqrt{\langle I(Q{\boldsymbol{^{\prime} }})\rangle }$, where *J*(**Q**, **Q′**) is the mutual intensity function (MIF) determined at the detector position. It is directly related to the statistical properties of the incident beam at the sample position by a two-dimensional Fourier transform

3 $$|J({\bf{Q}},{\bf{Q}}{\boldsymbol{^{\prime} }})|=|\iint {e}^{-i({\bf{Q}}{\boldsymbol{^{\prime} }}{\bf{r}}{\boldsymbol{^{\prime} }}-{\bf{Q}}{\bf{r}})}{J}_{in}({\bf{r}},{\bf{r}}{\boldsymbol{^{\prime} }})\,\,d{\bf{r}}\,\,d{\bf{r}}{\boldsymbol{^{\prime} }}|\,\mathrm{.}$$

Here, ${J}_{in}({\bf{r}},{\bf{r}}{\boldsymbol{^{\prime} }})=\langle {E}_{in}^{\ast }({\bf{r}}{\boldsymbol{^{\prime} }}){E}_{in}({\bf{r}})\rangle$ is the MIF function of the incoming field at the sample position, where *E*_*in*_(***r***) is the complex amplitude of the incident beam. In our experiment, the beam was focused on the sample by CRLs with an aperture larger than the incoming x-ray beam. As such, the coherence properties of the beam were preserved from the sample to detector position^57^.

It is important to note that the contrast function *ζ*_2_(*σ*_*ω*_) in Eq. (2) is defined by the longitudinal coherence properties of the beam, which are preserved between the sample and detector positions. Thus, it links the pulse duration of the beam to the coherence time of the monochromatized beam incident on the sample.

### Spatial degree of coherence and contrast values

Our experimental results also allowed us to determine the degree of spatial coherence *ζ*_*S*_ of LCLS radiation for hard x-rays. Similar to our previous work^12,13,27^, we obtained this value by applying the following relation

4 $${\zeta }_{S}=\frac{\int {|W({{\bf{r}}}_{{\bf{1}}},{{\bf{r}}}_{{\bf{2}}})|}^{2}d{{\bf{r}}}_{{\bf{1}}}d{{\bf{r}}}_{{\bf{2}}}}{{(\int \langle I({\bf{r}})\rangle d{\bf{r}})}^{2}}=\frac{\int {|\mu ({{\bf{r}}}_{{\bf{1}}},{{\bf{r}}}_{{\bf{2}}})|}^{2}\langle I({{\bf{r}}}_{{\bf{1}}})\rangle \langle I({{\bf{r}}}_{{\bf{2}}})\rangle d{{\bf{r}}}_{{\bf{1}}}d{{\bf{r}}}_{{\bf{2}}}}{{(\int \langle I({\bf{r}})\rangle d{\bf{r}})}^{2}}$$

and substituting values of |*μ*(***r***_**1**_, ***r***_**2**_)| obtained from the HBT interferometry analysis.

Evaluation of the contrast was performed based on their values determined from the main diagonal of the intensity correlation function *g*^(2)^(*x*, *x*). As a final value, the mean value of *g*^(2)^(*x*, *x*) over the region of FWHM of the averaged Bragg peak intensity 〈*I*(*x*)〉 was considered. For the contrast and spatial degree of coherence values determined for each Bragg peak and both samples as described above see Table II in Supplementary Material.

### Temporal properties of the beam

The contrast *ζ*_2_(*σ*_*ω*_) introduced in Eq. (2) can be determined from the values of the intensity correlation function along the main diagonal *g*^(2)^(*x*, *x*) (Fig. 3). Assuming a Gaussian Schell-model pulsed source^58^ (see Supplementary Material for details), the contrast function *ζ*_2_(*σ*_*ω*_) can be expressed as

5 $${\zeta }_{2}({\sigma }_{\omega })=\frac{1}{\sqrt{1+\mathrm{4(}{T}_{rms}{\sigma }_{\omega }{)}^{2}}}\,,$$

where *T*_*rms*_ is an effective pulse duration (r.m.s.) before the monochromator. In the derivation of equation (5) it was assumed that the spectral width of the incoming radiation is much broader than the monochromator bandwidth and the spectral coherence width. These conditions are well-satisfied for the LCLS x-ray beam parameters and the monochromator used in the experiment. Inversion of equation (5) gives, for the FWHM of the pulse duration, (see Supplementary Material for details)

6 $$T=\frac{2.355}{2{\sigma }_{\omega }}\sqrt{\frac{1}{{[{\zeta }_{2}({\sigma }_{\omega })]}^{2}}-1}\,\mathrm{.}$$

An average number of longitudinal modes of radiation *M* is inversely proportional to the normalized dispersion of the energy distribution, which in our case coincides with the contrast function defined in Eq. (2) as *M* = 1/[*ζ*_2_(*σ*_*ω*_)]. Substituting this relation in Eq. (6) we obtain for the pulse duration

7 $$T=\frac{2.355}{2{\sigma }_{\omega }}\sqrt{{M}^{2}-1}\,\mathrm{.}$$

## Electronic supplementary material

Supplementary Material
