# Supplementary material for: Diffraction based Hanbury Brown and Twiss interferometry at a hard x-ray free-electron laser
Source: Sci Rep. 2018 Feb 2;8:2219. doi: 10.1038/s41598-018-19793-1 (PMC5797123; doi:10.1038/s41598-018-19793-1)
Supplement: Supplementary file 1 — Supplementary Material [file 41598_2018_19793_MOESM1_ESM.pdf]

Supplementary Material for
"Diffraction based Hanbury Brown and Twiss interferometry at a hard x-ray free-electron laser"

O. Yu. Gorobtsov, N. Mukharamova, S. Lazarev, M. Chollet, D. Zhu, Y. Feng, R.P. Kurta, J.-M. Meijer, G. Williams, M. Sikorski, S. Song, D. Dzhigaev, S. Serkez, A. Singer, A.V. Petukhov, and I. A. Vartanyants

1. Intensity correlation functions of a radiation field scattered from a crystal

We will consider a quasi-monochromatic x-ray beam $E_{in}(\boldsymbol{s})$ incident on a colloidal crystal in a shape of a thin slab of material (see Fig.1).

An exit surface wave from such a crystal can be written as

$$E_{ESW}(\boldsymbol{s}) = O(\boldsymbol{s})E_{in}(\boldsymbol{s})\,, \qquad (1)$$

where $O(\boldsymbol{s})$ is the so-called object function and $\boldsymbol{s}$ is the two-dimensional (2D) vector in transverse direction to the incoming beam at the position of the sample. For a thin slab of material an object function can be expressed through refractive index $n(\boldsymbol{s}, z)$ as [1]

$$O(\boldsymbol{s}) = e^{i\varphi(\boldsymbol{s})}\,, \qquad (2)$$

where $\varphi(\boldsymbol{s}) = k\int_0^{d(\boldsymbol{s})}(n(\boldsymbol{s}, z) - 1)\mathrm{d}z$ is the phase difference due to refraction. Here $d(\boldsymbol{s})$ is the crystal thickness at the position $\boldsymbol{s}$, $k = 2\pi/\lambda$ is the wave number and λ is the wavelength. At x-ray wavelength refractive index can be expressed as [1] $n(\boldsymbol{s}, z) = 1 - \delta(\boldsymbol{s}, z) + i\beta(\boldsymbol{s}, z)$, where $\delta(\boldsymbol{s}, z)$ is the real part of refractive index responsible for refraction and $\beta(\boldsymbol{s}, z)$ is the imaginary part responsible for absorption. Neglecting absorption and taking into account known relation between the real part of refractive index $\delta(\boldsymbol{s}, z)$ and electron density $\rho(\boldsymbol{s}, z)$ of the crystal [1] $\delta(\boldsymbol{s}, z) = \lambda r_e \rho(\boldsymbol{s}, z)/k$, where r_e is the classical electron radius, we obtain for the phase in the object function in Eq. (2)

$$\varphi(\boldsymbol{s}) = -\lambda r_e \int_0^{d(\boldsymbol{s})} \rho\,(\boldsymbol{s}, z)\mathrm{d}z. \qquad (3)$$

Taking into account that projection of a crystalline electron density is a periodic function we obtain that the object function in Eq. (2) is also 2D periodic function.

To determine distribution of the wavefield at the detector position we will propagate the exit surface wave to that position by performing convolution with the free space propagator $P_L(\boldsymbol{r})$

$$E_d(\boldsymbol{r}) = \int E_{ESW}\,(\boldsymbol{s})P_L(\boldsymbol{r} - \boldsymbol{s})\mathrm{d}\boldsymbol{s} = \int O\,(\boldsymbol{s})P_L(\boldsymbol{r} - \boldsymbol{s})E_{in}(\boldsymbol{s})\mathrm{d}\boldsymbol{s}\,, \qquad (4)$$

where $\boldsymbol{r}$ is the 2D coordinate at the detector position and L is the sample-detector distance. Propagator in Eq. (4) has a known form

$$P_L(\boldsymbol{r}-\boldsymbol{s}) = \frac{1}{i\lambda L}\exp\left[ik\frac{(\boldsymbol{r}-\boldsymbol{s})^2}{2L}\right]. \tag{5}$$

Taking now into account that the object function is a 2D periodic function it can be expanded into Fourier series as

$$O(\boldsymbol{s}) = \sum_{\boldsymbol{h}} O_{\boldsymbol{h}}\, e^{i\boldsymbol{h}\cdot\boldsymbol{s}}\,, \tag{6}$$

where $\boldsymbol{h}$ is the 2D reciprocal space vector and $O_{\boldsymbol{h}} = 1/V\int O\,(\boldsymbol{s})e^{-i\boldsymbol{h}\cdot\boldsymbol{s}}\mathrm{d}\boldsymbol{s}$ are the Fourier coefficients of the expansion. Substituting now this expansion in Eq. (4) and considering scattering in the vicinity of a selected Bragg peak $\boldsymbol{h}$ we obtain

$$E_h(\boldsymbol{s}) = O_{\boldsymbol{h}}\int e^{i\boldsymbol{h}\cdot\boldsymbol{s}}\,P_L(\boldsymbol{r}-\boldsymbol{s})E_{in}(\boldsymbol{s})\mathrm{d}\boldsymbol{s}\,. \tag{7}$$

Using *Fraunhofer* far-field expression ($D^2/(\lambda L) \gg 1$, where D is the size of the beam at the sample position) of the propagator $P_L(\boldsymbol{r}-\boldsymbol{s}) \simeq exp(-iq_{\boldsymbol{r}}\cdot\boldsymbol{s})$, where $q_{\boldsymbol{r}} = k\boldsymbol{r}/L$ we obtain from Eq. (7)

$$E_h^{FF}(\boldsymbol{Q}) = O_{\boldsymbol{h}}\int e^{-i\boldsymbol{Q}\cdot\boldsymbol{s}}\,E_{in}(\boldsymbol{s})\mathrm{d}\boldsymbol{s}\,, \tag{8}$$

where $\boldsymbol{Q} = q_{\boldsymbol{r}} - \boldsymbol{h}$ is the momentum transfer vector calculated from the reciprocal space vector $\boldsymbol{h}$. For the intensity of the scattered field in the far-field we have

$$I_h^{FF}(\boldsymbol{Q}) = |E_h(\boldsymbol{Q})|^2 = |O_{\boldsymbol{h}}|^2\int\int e^{-i\boldsymbol{Q}\cdot(s-s')}\,E_{in}^*(\boldsymbol{s}')E_{in}(\boldsymbol{s})\mathrm{d}\boldsymbol{s}\mathrm{d}\boldsymbol{s}'\,. \tag{9}$$

In *Fresnel* (near-field) regime we can not use expansion expression for the propagator and we have for the scattered amplitude

$$E_h^{NF}(\boldsymbol{Q}) = O_{\boldsymbol{h}}e^{i\phi_r}\int e^{-i\boldsymbol{Q}\cdot\boldsymbol{s}}\,\tilde{E}_{in}(\boldsymbol{s})\mathrm{d}\boldsymbol{s}\,, \tag{10}$$

where we introduced the phase $\phi_r = k\boldsymbol{r}^2/(2L)$ and defined a new amplitude

$$\tilde{E}_{in}(\boldsymbol{s}) = e^{i\phi_s}E_{in}(\boldsymbol{s})\,, \tag{11}$$

where the phase ϕ_s is defined as $\phi_s = k\boldsymbol{s}^2/(2L)$. For intensity in the near-field we have

$$I_h^{NF}(\boldsymbol{Q}) = |E_h^{NF}(\boldsymbol{Q})|^2 = |O_{\boldsymbol{h}}|^2\int\int e^{-i\boldsymbol{Q}\cdot(s-s')}\,\tilde{E}_{in}^*(\boldsymbol{s}')\tilde{E}_{in}(\boldsymbol{s})\mathrm{d}\boldsymbol{s}\mathrm{d}\boldsymbol{s}'\,. \tag{12}$$

As we can see expressions for the scattered intensities around selected Bragg peak coincide in the far-field and near-field conditions with the change of the incoming wavefield expression to one given in Eq. (11). As soon as the difference between two cases is in the constant phase factor it would not influence statistical characteristics of the scattered field. In the following we will use far-field expression (9) keeping in mind that Fresnel conditions can be matched by the

substitution given in Eq. (11).

We will now evaluate intensity correlation function at the detector position in the vicinity of the selected Bragg reflection $\boldsymbol{h}$

$$g^{(2)}(\boldsymbol{Q},\boldsymbol{Q}') = \frac{< I(\boldsymbol{Q}) I(\boldsymbol{Q}') >}{< I(\boldsymbol{Q}) >< I(\boldsymbol{Q}') >}, \tag{13}$$

where momentum transfer vectors $\boldsymbol{Q}$ and $\boldsymbol{Q}'$ are centered at reflection $\boldsymbol{h}$ and related to the spatial coordinates at the detector position by $\boldsymbol{Q} = k\boldsymbol{u}/L$ and $\boldsymbol{Q}' = k\boldsymbol{u}'/L$. Averaging here is denoted by the brackets $<\ldots>$ and is performed over many realizations of the field. Substituting here expression for the intensity (9) we have for the nominator

$$< I(\boldsymbol{Q}) I(\boldsymbol{Q}') >=$$
$$= |O_{\boldsymbol{h}}|^4 \int \iiint e^{-i\boldsymbol{Q}\cdot(s-s')-\boldsymbol{Q}'\cdot(s''-s''')} \langle E_{in}^*(\boldsymbol{s}') E_{in}(\boldsymbol{s}) E_{in}^*(\boldsymbol{s}''') E_{in}(\boldsymbol{s}'')\rangle \mathrm{d}\boldsymbol{s}\mathrm{d}\boldsymbol{s}'\mathrm{d}\boldsymbol{s}''\mathrm{d}\boldsymbol{s}'''. \tag{14}$$

Assuming that the incoming radiation is chaotic and therefore obeys Gaussian statistics we can use Gaussian moment theorem

$$\begin{aligned}\langle E_{in}^*(\boldsymbol{s}') E_{in}(\boldsymbol{s}) E_{in}^*(\boldsymbol{s}''') E_{in}(\boldsymbol{s}'')\rangle &= \langle E_{in}^*(\boldsymbol{s}') E_{in}(\boldsymbol{s})\rangle\langle E_{in}^*(\boldsymbol{s}''') E_{in}(\boldsymbol{s}'')\rangle \\ &+ \langle E_{in}^*(\boldsymbol{s}') E_{in}(\boldsymbol{s}'')\rangle\langle E_{in}^*(\boldsymbol{s}''') E_{in}(\boldsymbol{s})\rangle. \end{aligned} \tag{15}$$

Substituting now this expression in Eq. (14) we obtain for the nominator

$$\langle I(\boldsymbol{Q}) I(\boldsymbol{Q}')\rangle = \langle I(\boldsymbol{Q})\rangle\langle I(\boldsymbol{Q}')\rangle + |J(\boldsymbol{Q},\boldsymbol{Q}')|^2, \tag{16}$$

where $|J(\boldsymbol{Q},\boldsymbol{Q}')|$ is the absolute value of the mutual intensity function (MIF) defined at the detector position and related to the MIF of the incoming field $J_{in}(\boldsymbol{s},\boldsymbol{s}') = \langle E_{in}^*(\boldsymbol{s}') E_{in}(\boldsymbol{s})\rangle$ by the following relation

$$|J(\boldsymbol{Q},\boldsymbol{Q}')|^2 = \left|\iint e^{-i(\boldsymbol{Q}'\boldsymbol{s}'-\boldsymbol{Q}\boldsymbol{s})} J_{in}(\boldsymbol{s},\boldsymbol{s}') d\boldsymbol{s} d\boldsymbol{s}'\right|^2. \tag{17}$$

Finally, we have for the normalized intensity correlation function (13)

$$g^{(2)}(\boldsymbol{Q},\boldsymbol{Q}') = \frac{\langle I(\boldsymbol{Q}) I(\boldsymbol{Q}')\rangle}{\langle I(\boldsymbol{Q})\rangle\langle I(\boldsymbol{Q}')\rangle} = 1 + |\mu(\boldsymbol{Q},\boldsymbol{Q}')|^2, \tag{18}$$

where

$$\mu(\boldsymbol{Q},\boldsymbol{Q}') = \frac{J(\boldsymbol{Q},\boldsymbol{Q}')}{\sqrt{\langle I(\boldsymbol{Q})\rangle}\sqrt{\langle I(\boldsymbol{Q}')\rangle}} \tag{19}$$

is the normalized spectral degree of coherence.

Taking now into account that we have a finite bandwidth of radiation incoming from the monochromator we have for the intensity correlation function

$$g^{(2)}(\boldsymbol{Q},\boldsymbol{Q}') = 1 + \zeta_2(\sigma_\omega)|\mu(\boldsymbol{Q},\boldsymbol{Q}')|^2\,, \tag{20}$$

where $\zeta_2(\sigma_\omega)$ is the contrast function which strongly depends on the radiation bandwidth σ_ω and averaged pulse duration T. We will now evaluate this function in the next section.

2. Determination of the pulse duration from the intensity interferometry

In the HBT interferometry the contrast function $\zeta_2(\sigma_\omega)$ for a cross-spectral pure chaotic radiation can be defined as [2-3]

$$\zeta_2(\sigma_\omega) = \frac{\iint_{-\infty}^{\infty} |T(\omega_1)|^2 |T(\omega_2)|^2 |W(\omega_1,\omega_2)|^2 \mathrm{d}\omega_1 \mathrm{d}\omega_2}{\left[\int_{-\infty}^{\infty} |T(\omega)|^2 S(\omega)\mathrm{d}\omega\right]^2}, \tag{21}$$

where $|\mathrm{T}(\omega)|^2$ is the monochromator transmission function, $W(\omega_1,\omega_2)$ is the cross spectral density function in the spectral domain, and $S(\omega) = W(\omega,\omega)$ is the spectral density function.

We will assume in the following that monochromator transmission function is described by a Gaussian function with the r.m.s. width σ_ω

$$|T(\omega)|^2 = \exp\left[\frac{\omega^2}{2\sigma_\omega^2}\right] \tag{22}$$

and pulsed x-ray radiation incoming on the monochromator can be approximated by a Gaussian Schell-model beam giving for the cross spectral density function [4]

$$W(\omega_1,\omega_2) = W_0 \exp\left[-\frac{(\omega_1-\omega_0)^2 + (\omega_2-\omega_0)^2}{4\Omega^2} - \frac{(\omega_1-\omega_2)^2}{2\Omega_c^2}\right], \tag{23}$$

where W_0 is the normalization constant. Here ω_0 is the central pulse frequency, Ω is the spectral width, and Ω_c is the spectral coherence width. It can be shown [4] that these parameters can be related to the r.m.s. values of the pulse duration T_{rms} and coherence time T_c of the pulse before monochromator as [4]

$$\Omega^2 = \frac{1}{T_c^2} + \frac{1}{4T_{rms}^2}\;;\; \Omega_c = \frac{T_c}{T_{rms}}\Omega\,. \tag{24}$$

Now substituting Eqs. (22 - 24) into the expression for the contrast function Eq. (21) and performing integration we obtain

$$\zeta_2(\sigma_\omega) = \frac{2C}{\sqrt{4A^2 - B^2}}, \tag{25}$$

where

$$A = \frac{1}{2\sigma_\omega^2} + \frac{1}{2\Omega^2} + \frac{1}{\Omega_c^2}\;; B = \frac{2}{\Omega_c^2}\;; C = \frac{1}{2\sigma_\omega^2} + \frac{1}{2\Omega^2}\,. \tag{26}$$

This is the general expression for the contrast function for arbitrary values of all frequencies introduced in this expression. Now taking into account that in the conditions of our experiment

at LCLS the monochromator bandwidth σ_ω and spectral coherence width Ω_c were much narrower than the spectral width Ω ($\sigma_\omega, \Omega_c \ll \Omega$) we obtain for parameters (26) the following approximate expression

$$A \simeq \frac{1}{2\sigma_\omega^2} + \frac{1}{\Omega_c^2}\,; B = \frac{2}{\Omega_c^2}\,; C \simeq \frac{1}{2\sigma_\omega^2}\,. \tag{27}$$

Substituting these values in expression (25) we obtain for the contrast function

$$\zeta_2(\omega) = \frac{\Omega_c}{\sqrt{\Omega_c^2 + 4\sigma_\omega^2}} = \frac{1}{\sqrt{1 + 4(\sigma_\omega/\Omega_c)^2}}\,. \tag{28}$$

Taking now into account that in the conditions of our experiment at LCLS coherence time of radiation before the monochromator was much shorter than the pulse duration ($T_c \ll T_{rms}$) we obtain from Eqs. (24) for the pulse duration

$$T_{rms} \simeq \frac{1}{\Omega_c}\,. \tag{29}$$

Substituting this expression in Eq. (28) we obtain for the contrast function the following relation

$$\zeta_2(\omega) = \frac{\Omega_c}{\sqrt{\Omega_c^2 + 4\sigma_\omega^2}} = \frac{1}{\sqrt{1 + 4(T_{rms}\sigma_\omega)^2}}\,. \tag{30}$$

that was used in the main text of the manuscript for the analysis. In two limiting cases $T_{rms}\sigma_\omega \ll 1$ and $T_{rms}\sigma_\omega \gg 1$ we obtain from equation (30) for the contrast function: $\zeta_2(\sigma_\omega) \simeq 1 - 2(T_{rms}\sigma_\omega)^2$ in the first case and $\zeta_2(\sigma_\omega) \simeq 1/[2(T_{rms}\sigma_\omega)]$ in the second. The first case corresponds to nearly Fourier limited radiation and the second one to rather incoherent (in time-domain) radiation.

Expression (30) can be inverted to determine averaged pulse duration of the x-ray radiation before the monochromator. For the FWHM of the pulse duration we finally have

$$T = 2.355T_{rms} = \frac{2.355}{2\sigma_\omega}\sqrt{\frac{1}{[\zeta_2(\sigma_\omega)]^2} - 1}\,. \tag{31}$$

3. Additional experimental results

Here we present additional figures demonstrating our experimental results. Projections of the averaged intensity on the both horizontal and vertical axes (shown in Fig. 2) reveal presence of the small subpeaks due to the defect structure of the colloidal crystal. Cross sections of the intensity correlation function along the white line in Fig. 3 in the main text (see Fig. 3) demonstrate a relatively flat region in the center and a steep slope after that.

4. Simulation of the intensity correlation functions

The model used for simulations in this work was first introduced in Ref. [3]. In this model, the X-ray beam is assumed to consist of several statistically independent Gaussian Schell-model beams with the total complex field amplitude

$$E_\Sigma(\boldsymbol{r},\omega) = \sum_{i=1}^{N} E_i\,(\boldsymbol{r},\omega)\,, \tag{32}$$

where $E_i(\boldsymbol{r},\omega)$ is a complex amplitude of a single beam. Since all beams are statistically independent, the total spectral cross-correlation function $W_\Sigma^{(2)}(\boldsymbol{r_1},\omega_1,\boldsymbol{r_2},\omega_2)$ and spectral density $S_\Sigma(\boldsymbol{r},\omega)$ can be expressed as

$$W_\Sigma^{(2)}(\boldsymbol{r_1},\omega_1,\boldsymbol{r_2},\omega_2) = \sum_{i=1}^{N} J_i\,(\boldsymbol{r_1},\boldsymbol{r_2}) W_i(\omega_1,\omega_2)\,, \tag{33}$$

$$S_\Sigma(\boldsymbol{r},\omega) = \sum_{i=1}^{N} I_i\,(\boldsymbol{r}) S_i(\omega)\,. \tag{34}$$

Intensity cross-correlation function is than calculated similar to Ref. [3]

$$g_\Sigma^{(2)}(\boldsymbol{r_1},\boldsymbol{r_2}) = 1 + \\ + \frac{\sum_{i,j=1}^{N} J_i(\boldsymbol{r_1},\boldsymbol{r_2}) J_j^*(\boldsymbol{r_1},\boldsymbol{r_2}) \iint_{-\infty}^{\infty} |T(\omega_1)|^2 |T(\omega_2)|^2 W_i(\omega_1,\omega_2) W_j^*(\omega_1,\omega_2) \mathrm{d}\omega_1 \mathrm{d}\omega_2}{\sum_{k,l=1}^{N} I_k(\boldsymbol{r_1}) I_l(\boldsymbol{r_2}) \int_{-\infty}^{\infty} |T(\omega_1)|^2 S_k(\omega_1) \mathrm{d}\omega_1 \int_{-\infty}^{\infty} |T(\omega_2)|^2 S_l(\omega_2) \mathrm{d}\omega_2} \tag{35}$$

The model for simulating the fluctuating detector background was also introduced in Ref. [3]. The total intensity can be represented as

$$I(x) = I_0(x) + I_B(x)\,, \tag{36}$$

where $I_0(x)$ is the intensity of the beam and $I_B(x)$ is the background intensity. The background signal is assumed to be statistically independent from the beam intensity fluctuations. It is then possible to express the normalized intensity correlation function modified by fluctuating background as

$$g^{(2)}(x_1,x_2) = \\ = \frac{\langle I_0(x_1) I_0(x_2)\rangle + \langle I_B(x_1) I_B(x_2)\rangle + \langle I_0(x_1)\rangle\langle I_B(x_2)\rangle + \langle I_B(x_1)\rangle\langle I_0(x_2)\rangle}{(\langle I_0(x_1)\rangle + \langle I_B(x_1)\rangle)(\langle I_0(x_2)\rangle + \langle I_B(x_2)\rangle)}, \tag{37}$$

where the ensemble average $\langle I_0(x_1) I_0(x_2)\rangle = g_\Sigma^{(2)}(x_1,x_2) \cdot \langle I_0(x_1)\rangle\langle I_0(x_2)\rangle$. The background average intensity and intensity correlation function are assumed to have the form

$$\langle I_B(x)\rangle = C\,, \tag{38}$$

$$\langle I_B(x_1) I_B(x_2)\rangle = C^2(1 + A\delta_{x_1,x_2})\,, \tag{39}$$

where $C << max(I_0)$ and the background signal is therefore not significant in the center of

the beam.

The final expression that was used for modeling as it follows from Eqs. (35 - 39) has the form

$$g^{(2)}(x_1, x_2) = \\ = \frac{g_\Sigma^{(2)}(x_1, x_2)\langle I_\Sigma(x_1)\rangle\langle I_\Sigma(x_2)\rangle + C^2(1 + A\delta_{x_1,x_2}) + C\langle I_\Sigma(x_1)\rangle + C\langle I_\Sigma(x_2)\rangle}{(\langle I_\Sigma(x_1)\rangle + C)(\langle I_\Sigma(x_2)\rangle + C)}. \quad (40)$$

In simulations we used two models (see Table 1): one in the horizontal direction consisting of a single beam with the size (r.m.s.) 1.6 mm and coherence length 10 mm and second one in the vertical direction consisting of two beams shifted by 1.5 mm and with the relative intensity of 10%. The background level was considered to be 2% of the total intensity in both cases and parameter A in Eq.(8) was taken as $A = 0.125$. All further details of all parameters in both models are listed in Table 1.

5. Spatial degree of coherence and contrast values

The contrast and spatial degree of coherence values determined for each Bragg peak and both samples as described in Methods section of the main text are listed in Table II.

6. Mode analysis and electron bunch energy filtering

Jitter in the energy of the electron bunch introduces additional problem for the analysis of the monochromator filtered radiation. If electron bunch energy of a pulse is significantly different from an average, the central wavelength of the pulse is too far removed from the monochromator transmittance band.

In such a case pulse intensity after monochromator will be significantly reduced, affecting observed statistics. This is clearly observed in Fig. 4, where the distribution of pulse intensities and corresponding electron bunch energies is shown.

Therefore, it is important to filter the collected pulses by bunch energy. The filtering was performed by choosing only the pulses for which

$$E_{el} - \langle E_{el}\rangle < \sigma_{E_{el}}/2\,, \quad (41)$$

where E_{el} is the electron bunch energy and $\sigma_{E_{el}}$ is the *r.m.s.* of the energy dispersion (see Fig. 4 for the region considered for the following analysis). Around $50{,}000$ pulses were left in each run after the filtering. The difference in the intensity distribution due to filtering can be seen in Fig. 5, where the histogram of the integrated intensity from the Bragg peak in the diffraction pattern before and after the electron bunch filtering is shown. The number of modes

is clearly underestimated without filtering.

The pulse duration was also determined by using the mode analysis of the radiation as suggested in Ref. [5]. According to this approach an average number of modes of radiation M is inversely proportional to the normalized dispersion of the energy distribution, that in our case coincide with the contrast function defined in Eq. (20) $M = 1/[\zeta_2(\sigma_\omega)]$. Substituting this relation in Eq. (30) we obtain for the pulse duration

$$T = \frac{2.355}{2\sigma_\omega}\sqrt{M^2 - 1}\,. \quad (42)$$

We determined the number of modes by fitting integrated intensity distribution at one of the Bragg peaks by Gamma distribution [5] (see Fig. 6). As a result, the number of longitudinal modes was $M \approx 2.3 \pm 0.1$ and reproducible between different runs. Substituting this number in Eq. (42) gives for the pulse duration 11.5 ± 0.5 fs.

References:

[1] Als-Nielsen, J. & McMorrow, D. *Elements of Modern X-ray Physics* (WILEY, 2-nd edition, 2011).

[2] Singer, A. *et al.* Hanbury Brown-Twiss Interferometry at a Free-Electron Laser. *Phys. Rev. Lett.* **111**, 034802 (2013). Erratum: *Phys. Rev. Lett.* **117**, 239903(E) (2016).

[3] Gorobtsov, O. Y. *et al.* Statistical properties of a free-electron laser revealed by Hanbury Brown-Twiss interferometry. *Phys. Rev. A* **95**, 023843 (2017).

[4] Lajunen, H., Vahimaa, P. & Tervo, J. Theory of spatially and spectrally partially coherent pulses. *J. Opt. Soc. Am. A* **22**, 1536-1545 (2005).

[5] Saldin, E., Schneidmiller, E. & Yurkov, M. Statistical properties of radiation from VUV and X-ray free electron laser. *Opt. Comm.* **148**, 383 - 403 (1998).

Table I. Beam parameters used in simulations

Simulation	Model I	Model II	
Beam number	1	1	2
Bandwidth, $2.355\sigma_\omega/\omega$	$4.4\cdot10^{-5}$	$4.4\cdot10^{-5}$	
Relative intensity	1	1	0.1
Beam position, x_0 (mm)	0	-	-
y_0 (mm)	-	0	-1.5
Beam size (rms), σ (mm)	1.6	1.4	0.5
Transverse coherence length, ξ (mm)	10	8	10
Central frequency, ω_0 (as^{-1})	12.6	12.6	12.6
Spectral width, Ω (fs^{-1})	6.3	6.3	6.3
Spectral coherence width, Ω_c (fs^{-1})	0.2	0.2	1.1

Table II. Contrast values ζ_2 and spatial degree of coherence ζ_S determined at different Bragg peaks and both crystals for each direction

Crystal	Sample 1 (PS 160 nm)				Sample 2 (PS 420 nm)			
Peak number	1	2	3	4	1	2	3	4
ζ_2, x	0.41	0.40	0.41	0.40	0.41	0.43	0.41	0.43
ζ_2, y	0.42	0.41	0.43	0.41	0.44	0.46	0.45	0.41
ζ_S, x	0.92	0.94	0.94	0.93	0.93	0.89	0.95	0.88
ζ_S, y	0.90	0.91	0.90	0.91	0.87	0.82	0.85	0.93

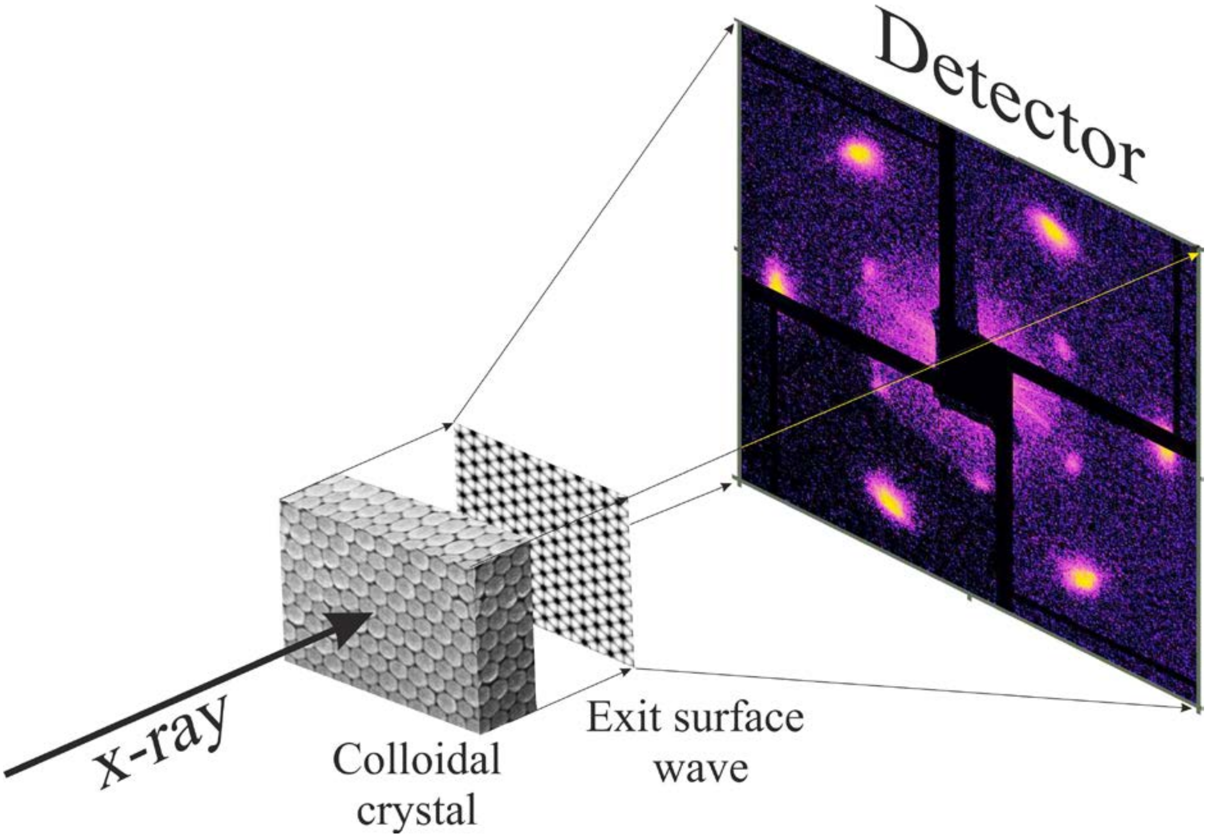

Fig. 1. Scheme of diffraction experiment on a colloidal crystal performed at LCLS. An x-ray beam from the LCLS is incoming on a thin colloidal crystal film, just behind a film an exit surface wave is formed that is propagating in free space towards detector position.

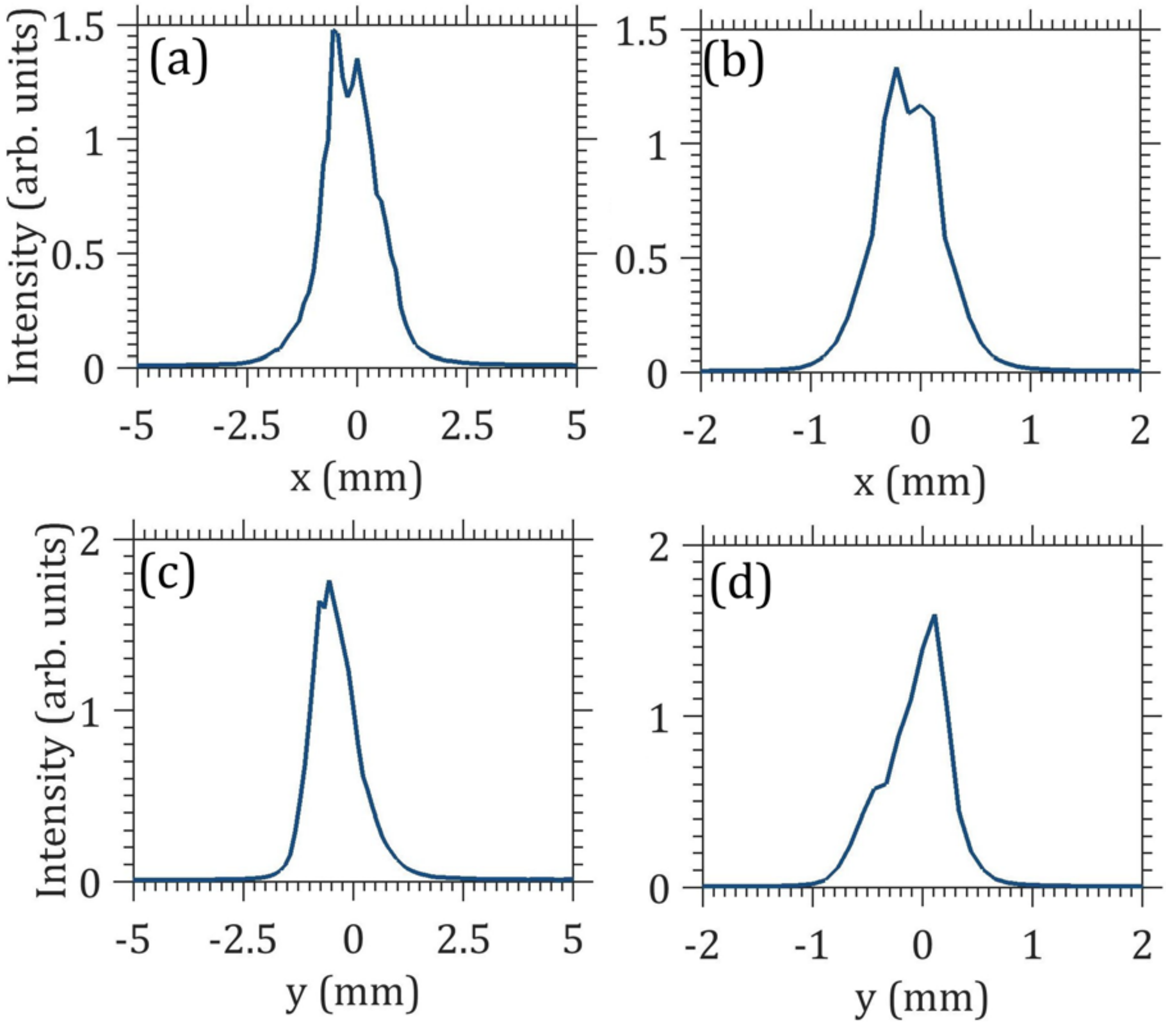

Fig. 2. Projections of the averaged intensity. (a, c) Horizontal and vertical projections for the sample 1. (b, d) Horizontal and vertical projections for the sample 2.

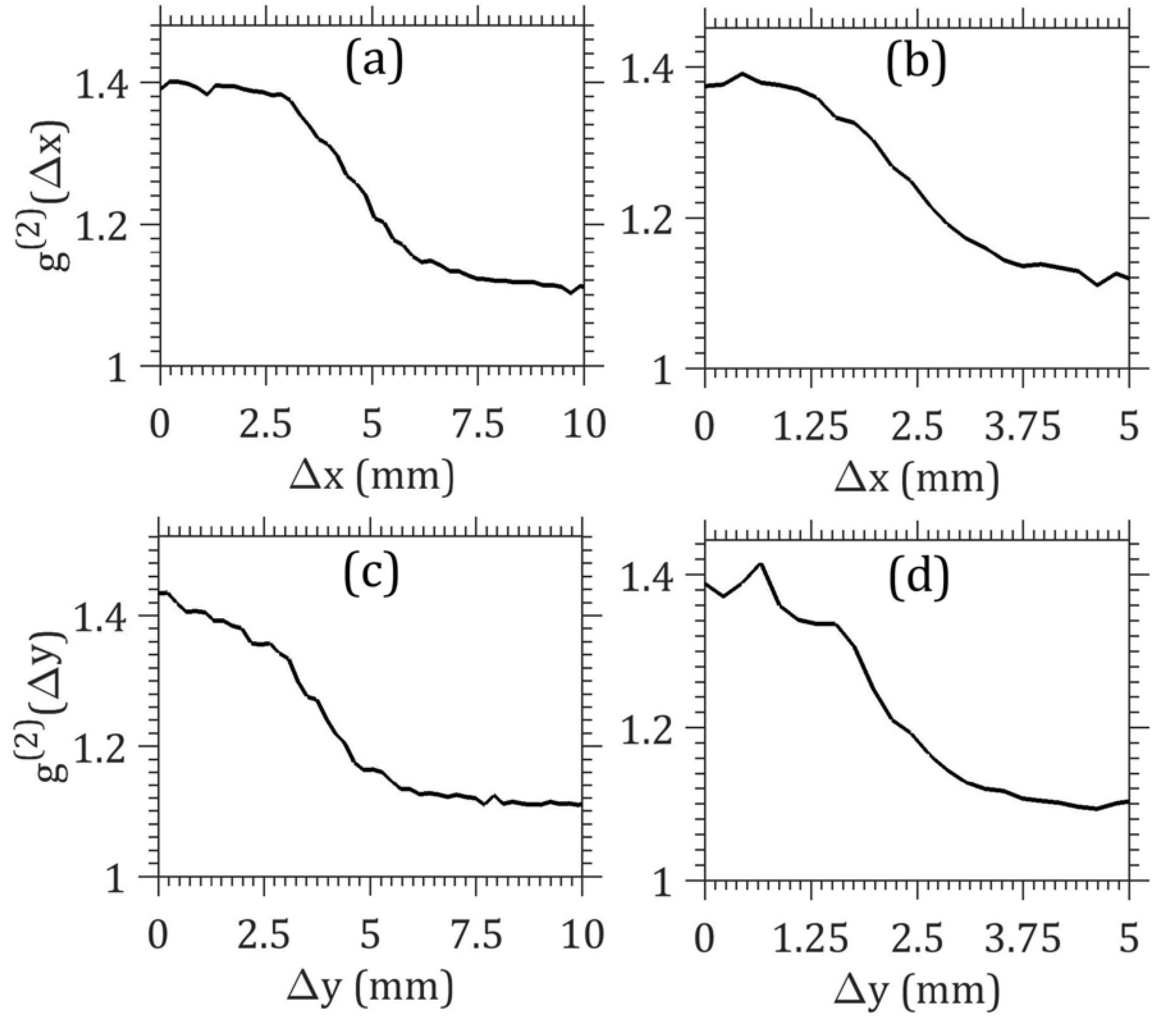

Fig. 3. Intensity correlation functions $g^{(2)}(\Delta x)$ and $g^{(2)}(\Delta y)$ taken along the diagonal shown in Fig. 3 (c, f) of the main text as a white line for sample 1 (a, c) and sample 2 (b, d).

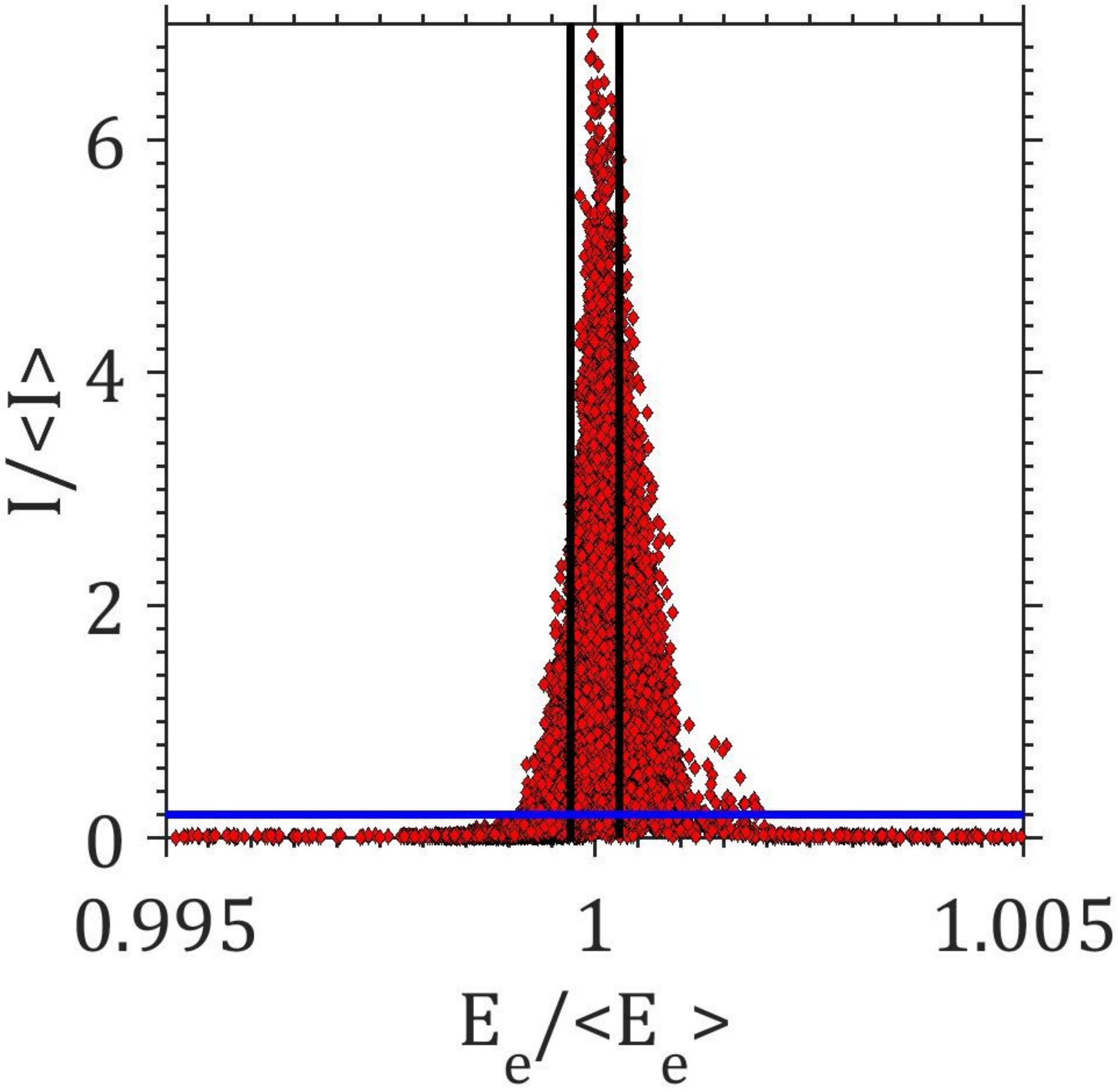

Fig. 4. Distribution of normalized pulse intensities and corresponding electron bunch energies for the run with the sample 1. Black lines show the limits of the filtered region. Blue line shows the cutoff used for the histogram fitting without filtering.

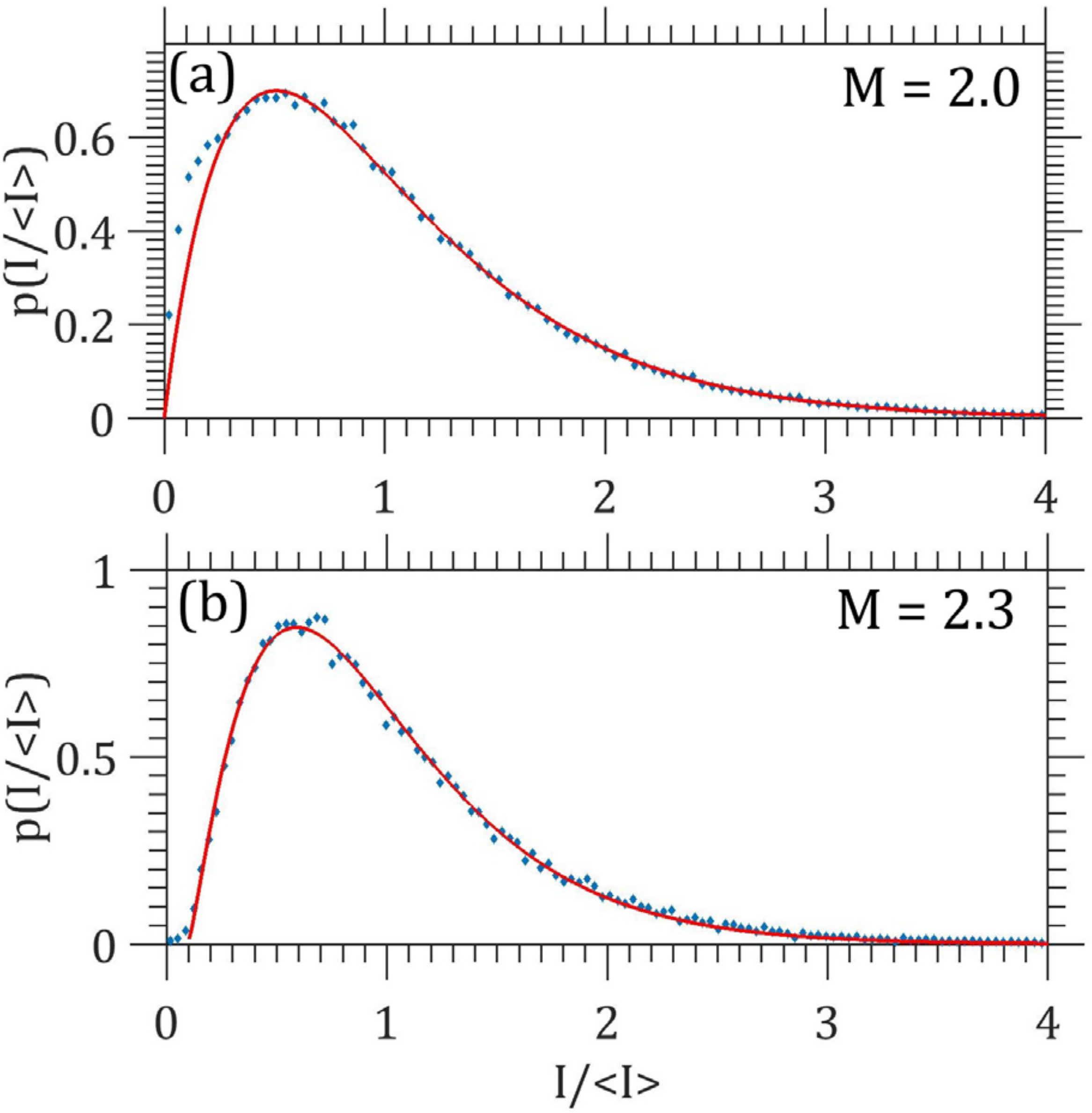

Fig. 5. Histogram of the integrated intensity (sample 1) from the Bragg peak 4 in the diffraction pattern before the electron bunch energy filtering (a) and after filtering (b).

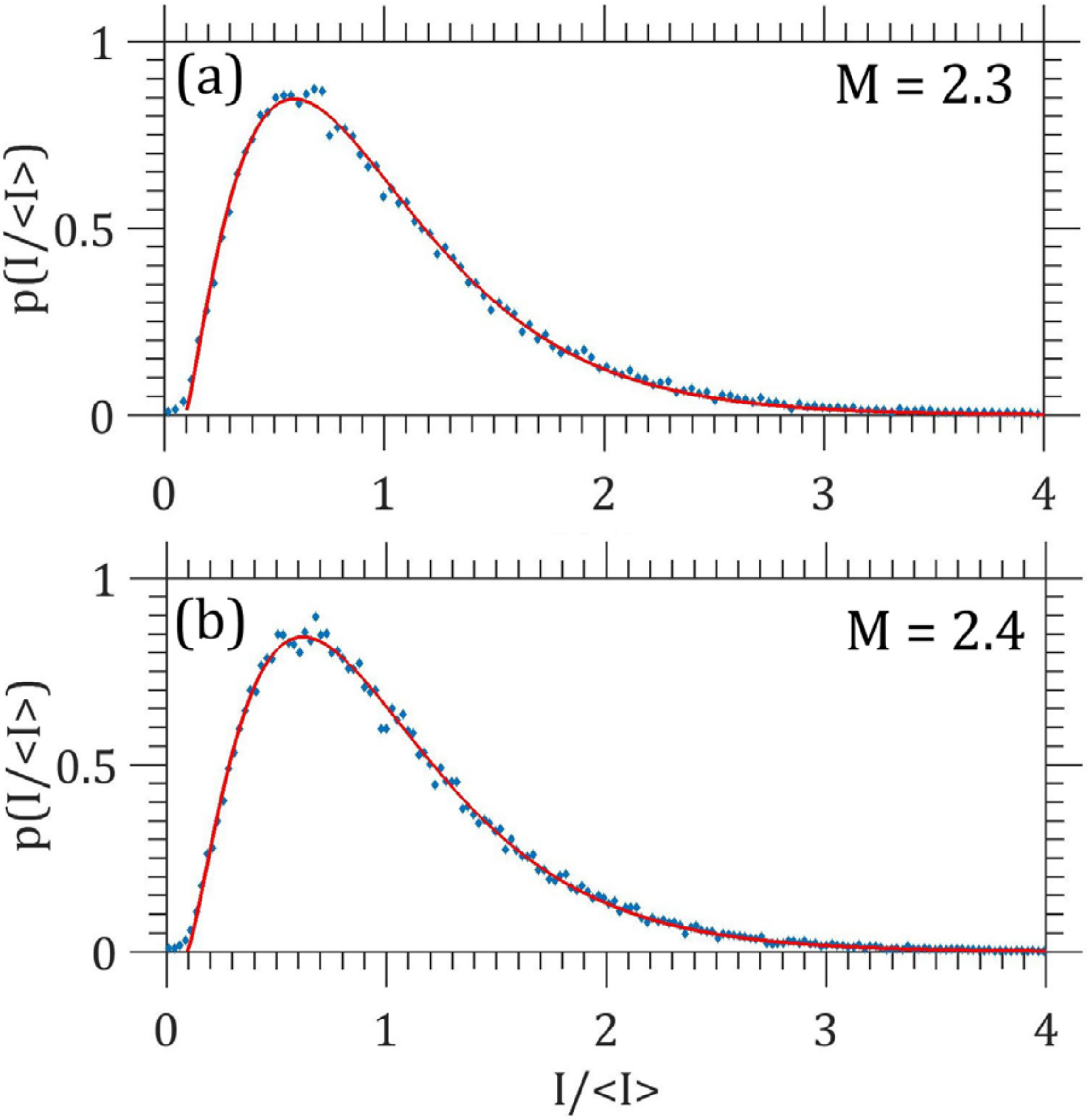

Fig. 6. Histogram of the integrated intensity (sample 1) from the Bragg peak 4 in the diffraction pattern (a) and from the intensity monitor after the monochromator (b).
